# Prognostic value and predictive biomarkers of phenotypes of tumour‐associated macrophages in colorectal cancer

**DOI:** 10.1111/sji.13137

**Published:** 2022-01-10

**Authors:** Yu Kou, Zhuoqun Li, Qidi Sun, Shengnan Yang, Yunshuai Wang, Chen Hu, Huijie Gu, Huangjian Wang, Hairong Xu, Yan Li, Baowei Han

**Affiliations:** ^1^ Jiangsu Key Laboratory of Integrated Traditional Chinese and Western Medicine for Prevention and Treatment of Senile Diseases Medical College of Yangzhou University Yangzhou China; ^2^ Department of Traditional Chinese Medicine Affiliated Hospital of Yangzhou University Yangzhou China; ^3^ Jiangsu Co‐Innovation Center for Prevention and Control of Important Animal Infectious Diseases and Zoonoses Yangzhou China; ^4^ Department of General Surgery Luoyang Central Hospital Affiliated of Zhengzhou University Luoyang China

**Keywords:** biomarker, colorectal cancer, tumour‐associated macrophages

## Abstract

**Background:**

The roles of different subtypes of tumour‐associated macrophages (TAMs) in predicting the prognosis of colorectal cancer (CRC) remain controversial. In this study, different subtypes of TAMs were investigated as prognostic and predictive biomarkers for CRC.

**Methods:**

Expressions of CD68, CD86 and CD163 were investigated by immunohistochemistry (IHC) and immunofluorescence (IF), and the correlation between the expression of CD86 and CD163 was calculated in colorectal cancer tissues from 64 CRC patients.

**Results:**

The results showed that high expressions of CD86^+^ and CD68^+^CD86^+^ TAMs as well as low expression of CD163^+^ and CD68^+^CD163^+^ TAMs were significantly associated with favourable overall survival (OS). The level of CD86 protein expression showed a negative correlation with CD163 protein expression. In addition, CD86 protein expression remarkably negatively correlated with tumour differentiation and tumour node metastasis (TNM) stage, while CD163 protein expression significantly positively correlated with tumour differentiation and tumour size. As an independent risk factor, high expression of CD86 TAMs had prominently favourable prognostic efficacy, while high expression of CD68^+^CD163^+^ TAMs had significantly poor prognostic efficacy.

**Conclusions:**

These results indicate that CD86^+^ and CD68^+^CD163^+^ TAMs as prognostic and predictive biomarkers for CRC.

## 1 INTRODUCTION

The latest epidemiological data discovered that colorectal cancer (CRC) is the third most common diagnosed cancer but the second leading cause of cancer‐related death globally, with some 1.8 million new patients and 0.861 million deaths in 2018.^1^ On the basis of data from Chinese National Cancer Center, CRC is the fourth highest incidence in women and the fifth highest incidence in men.^2^ In the last 20 years, even though implemented screenings for its early diagnosis and witnessed of available treatment modalities such as chemotherapy, targeted therapy and immunotherapy, CRC recurrence and metastasis remained very common and its mortality rate was still very high.^3^, ^4^, ^5^ Reports indicate that the 5‐year relative survival rate for CRC patients still remains around 65%, yet approximately 50% of CRC patients will eventually develop recurrence and metastasis.^4^ There is still a need for better prognostic and efficient biomarkers for early detection of CRC diagnosis and recurrence.

CRC was derived from chronic inflammatory tissues under the immune surveillance of tumour‐infiltrating immune cells. Tumour‐immune cells interaction is a significant territory of research in regard to prognosis in CRC. Cancer accelerating inflammation orchestrates a strong immune cell response and the tumour microenvironment (TME) are arising as crucial obstacles to the development of effective therapies.^6^, ^7^ Specifically, tumour‐associated macrophages (TAMs) are dominating immune cells in the TME and have been implicated in neoplastic progression, survival and metastatic dissemination in various solid tumours.^8^, ^9^ Meanwhile, TAMs also play a pivotal role in influencing the tumour activity and prognosis of CRC.^10^ However, clinically deciding the practical biologic relevance of TAMs has demonstrated to be difficult because TAMs cannot be assessed by standardized methods on haematoxylin/eosin (H&E) but immunohistochemistry (IHC).^11^ So many individual researches have uncertain results.

In general, TAMs have been divided into M1 and M2 subtypes to define their polarization status: M1 TAMs, which act in a tumour‐inhibiting manner by stimulating tumour immunity and suppressing tumour progression, and M2 TAMs, which act in a tumour‐promoting manner via promoting tumour cell invasion, motility and intravasation, enhancing angiogenesis, restraining the immune response and escaping tumour cell attack by natural killer and T cells.^12^, ^13^ Generally, different markers were used to identify TAMs in CRC, including the most common pan‐macrophage marker, CD68; M1 macrophage markers such as nitric oxide synthase (iNOS), CD86 and CD169; and M2 macrophage markers such as CD163, CD206 and CD204.^14^ Ohtaki et al used CD68 and CD204 as markers and researched TAMs in patients with lung cancer, with their results finding that CD204‐positive stromal TAMs but not CD68‐positive stromal TAMs are connected with tumour aggressiveness in lung cancer.^15^ In additional, ratio of CD206/CD68 TAMs is a better prognostic and predictive biomarker in patients with stage II colon cancer.^16^

However, little clinical evidence proved that TAMs were predictive biomarkers and prognostic risk factor in CRC. In the present research, we tested TAMs and M1/M2 subtypes that infiltrated in the tumour tissues of CRC cancer patients. We evaluated the prognostic and predictive accuracy of TAMs as biomarkers for post‐operative patients. We also analysed the clinicopathological characteristics of the CRC patients and risk factors to predict prognosis.

## 2 MATERIALS AND METHODS

### 2.1 Patients and specimens

To research the clinical and pathological significance of CD68, CD86 and CD163, we collected 64 specimens, diagnosed by clinical and histopathological evidence, from colorectal carcinoma patients, who were treated at the Luoyang Central Hospital Affiliated to Zhengzhou University (Luoyang, China) from March 2012 to March 2015. All patients were not treated in pre‐operative and were received post‐operative adjuvant chemotherapy. This research was approved by the Institutional Review Board and Human Ethics Committee at Luoyang Central Hospital Affiliated to Zhengzhou University, and agreed to use paraffin‐embedded colorectal tissue samples for the projected research acquired from all patients or their families. And all patients provided written and oral informed consent. All of the CRC patients had undergone curative resection, and the final pathological diagnosis was adenocarcinoma. The stage of CRC was confirmed according to the AJCC/UICC TNM staging system, 8th edition; and radical (R0) resection of the primary tumour. Furthermore, those patients were followed up after confirmed CRC on 15 April 2019. A total of 64 CRC samples were used for immunohistochemistry (IHC) analysis. Clinical and pathologic data, including age, gender tumour size, TNM stage, etc, were obtained from hospital medical records. Overall survival (OS) was defined as the time of confirmed CRC to the date of death or the latest follow up. Recurrence‐free survival (RFS) was defined as the time from the date of confirmed CRC to the first date of recurrence, or the date of the last follow up.

### 2.2 Immunohistochemistry

Immunohistochemical staining was implemented on formalin‐fixed and paraffin‐embedded surgical tissue specimens. Slides were cut at 4 μm thickness. Paraffin sections were dewaxed in xylene and rehydrated in a gradient series of ethanol solutions. Endogenous peroxidases were blocked with 3% hydrogen peroxide for 10 minutes then fixed in 4% paraformaldehyde for 15 minutes. Sections were rinsed with phosphate‐buffered saline (PBS) 3 times for 5 minutes. Then, antigen retrieval was accomplished in citrate buffer (0.01 M) for 3 minutes at 95°C using a microwave oven. Slides were then incubated with primary antibodies against CD68 (Cat# sc‐20060, Santa Cruz,1:200), CD86 (Cat# sc‐28347, Santa Cruz,1:200) or CD163 (Cat# sc‐20066, Santa Cruz,1:200) overnight at 4°C, followed by treatment with biotinylated secondary antibodies for 30 minutes at room temperature, then streptavidin‐biotin complex (SABC, Boster). Sections were reacted with diaminobenzidine (DAB, Boster) and counterstained with haematoxylin for nuclear staining. The IHC results were detected by 2 independent pathologists who were specialized and had no information of the patients’ clinical status. To quantify the immunostaining of CD68, CD86 and CD163, slides were imaged digitally with equal light exposure and assessed by Image Pro Plus (IPP). The immunostaining extent was scored by the percentage of positive cells (0‐100) using Image Pro Plus (IPP) and the immunostaining intensities were multiplied to produce an intensity score (0, 1, 2 and 3).

### 2.3 Immunofluorescence

According to the protocol described previously,^17^ the immunofluorescence assays were performed with M1 macrophage markers of CD68 and CD86 and M2 macrophage markers of CD68 and CD163 respectively. After being washed, the coverslips were incubated with the corresponding PE/FITC conjugated secondary antibodies m‐ IgGκBP‐PE (Cat# 516141, Santa Cruz, 1:200) and Alexa Fluor 488 AffiniPure Goat Anti‐Rabbit IgG (H+L) (FMS‐RBaf48801, FcMACS,1:200) for 30min, then counterstained with 5mg/ml DAPI for 20 min, after that, the images were detected by confocal microscopy (Leica, Jena, Germany).

### 2.4 Statistical analyses

All statistical analyses were executed with the SPSS version 19.0 software. The relativity analyses were employed using Pearson coefficient and P‐value between CD86 and CD163 staining scores. ROC analyses were performed to measure the cut points of IHC score for CD68, CD86 and CD163 in CRC tissue. The Chi‐squared test or Fisher's exact test was used to analyse the correlation between CD68, CD86 andCD163 and clinicopathological characteristics. The Kaplan‐Meier method with the log‐rank test was conducted to estimate survival difference and prognosis factors. For multivariate regression, only factors with *P* <.05 in the log‐rank univariate analyses were brought into the Cox's proportional hazard model. The survival outcomes were estimated with hazard ratio (HR) and its 95% confidence interval (CI). *P* <.05 was considered statistically significant.

## 3 RESULTS

### 3.1 Patient characteristics and follow‐up evaluation

In this study, of 78 CRC patients, 64 (82%) were included in the analysis. Of the 14 patients excluded, 6 (7.7%) had inadequate follow‐up date, 4 (5%) had undergone palliative resection, 2 (2.5%) had liver metastasis confirmed CRC and 2 (2.5%) had died not because of cancer. The detailed clinicopathological characteristics and high‐risk factors of CRC patients after radical resection are shown in Table 1 and Table 2. The median RFS of 64 CRC patients was 36 months, and 5‐year RFS rate of all enrolled patients was 14% (Figure 3A). Then, median OS of 64 CRC patients was 41 months, and the 5‐year survival rate of all enrolled patients was 26.4% (Figure 4A).

**TABLE 1:** Clinicopathological characteristics and log‐rank univariate analyses for RFS of the patients

Variables	Total patients (n)	X^2^ value	*P*‐value
Age			
≤60	26	0.016	.898
>60	38		
Sex			
Male	35	0.476	.490
Female	29		
Tumour location			
Colon	28	0.096	.757
Rectal	36		
Tumour size(cm)			
<3	12	8.217	.004
≥3	52		
Differentiation			
Well/moderate	48	6.495	.011
Poor/undifferentiated	16		
T stage			
T1‐T2	20	5.733	.017
T3‐T4	44		
TNM stage			
Ⅰ	18	9.509	.023
Ⅱ	14		
Ⅲ	27		
Ⅳ	5		
Lymph node metastasis			
No	33	5.534	.019
Yes	31		
Distant metastasis			
No	60	3.460	.063
Yes	4		
CD68 protein expression			
Low	47	0.286	.593
High	17		
CD86 protein expression			
Low	54	9.993	.002
High	10		
CD163 protein expression			
Low	26	12.064	.001
High	38		
Co‐expression of CD68 and CD86			
Both high	8	6.489	.011
Others	56		
Co‐expression of CD68 and CD163			
Both high	10	5.836	0.016
Others	54		

Note

*P*‐values were obtained by log‐rank test.

**TABLE 2:** Clinicopathological characteristics and log‐rank univariate analyses for OS of the patients

Variables	Total patients(n)	X^2^ value	*P*‐value
Age			
≤60	26	0.051	.822
>60	38		
Sex			
Male	35	0.583	.445
Female	29		
Tumour location			
Colon	28	0.122	.727
Rectal	36		
Tumour size (cm)			
<3	12	8.958	.003
≥3	52		
Differentiation			
Well/moderate	48	5.535	.019
Poor/undifferentiated	16		
T stage			
T1‐T2	20	7.025	.008
T3‐T4	44		
TNM stage			
Ⅰ	18	12.422	.006
Ⅱ	14		
Ⅲ	27		
Ⅳ	5		
Lymph node metastasis			
No	33	7.695	.006
Yes	31		
Distant metastasis			
No	60	3.430	.064
Yes	4		
CD68 protein expression			
Low	47	0.374	.541
High	17		
CD86 protein expression			
Low	54	10.199	.001
High	10		
CD163 protein expression			
Low	26	12.097	.001
High	38		
Co‐expression of CD68 and CD86			
Both high	8	6.518	.011
Others	56		
Co‐expression of CD68 and CD163			
Both high	10	5.201	.023
Others	54		

Note

*P*‐values were acquired by log‐rank test.

### 3.2 The biomarkers of TAM protein expressed in human CRC

**FIGURE 1:**
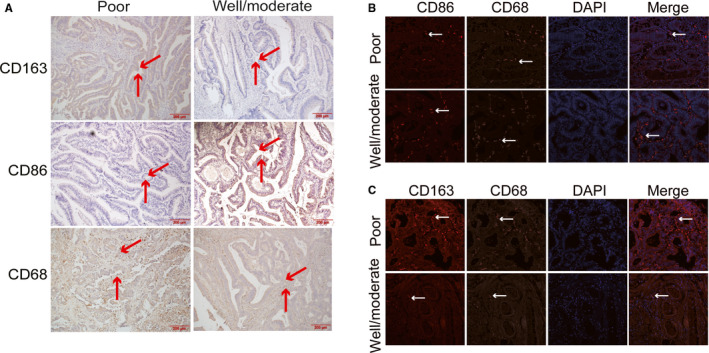
Detection of CD68, CD163 and CD86 using immunohistochemical staining and multiplex quantitative immunofluorescence in poor differentiated and well/moderate differentiated colorectal cancer. A, Representative immunohistochemical staining images of CD68, CD86 and CD163. B, Representative fluorescence images showing the estimate of M1 macrophage in colorectal cancer tissues by simultaneous staining of DAPI (blue channel), CD68 (Alexa Fluor 488, green channel) and CD86 (PE, red channel). C, Representative fluorescence images showing the estimate of M2 macrophage in colorectal cancer tissues by simultaneous staining of DAPI (blue channel), CD68 (Alexa Fluor 488, green channel) and CD163 (PE, red channel)

**FIGURE 2:**
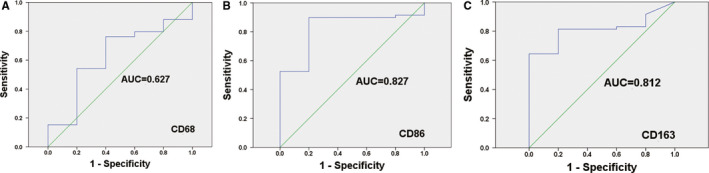
ROC statistics were used to detect the cut points of the IHC score for CD68, CD86 and CD163. A, CD68, B, CD86 and C, CD163 in colorectal cancer samples

CD68 was chosen as a marker for most common pan‐macrophage, CD86 as a marker for M1‐like macrophages and CD163 as a marker for M2‐like phenotype. In order to examine the expression of CD68, CD86 and CD163 in CRC, we performed IHC and IF analyses of 64 CRC specimens (Figure 1). All CRC tissue slides were digitally imaged and evaluated by Image Pro Plus. ROC statistics were employed to evaluate the cut point of the IHC score for TAMs as shown in Figure 2. Therefore, for CD68 protein expression, the IHC scores of ≥90.75 was defined as high and <90.75 was defined as low. For CD86 protein expression, the IHC scores of ≥87.52 was defined as high and <87.52 was defined as low. For CD163 protein expression, the IHC scores of ≥27.89 was defined as high and <27.89 was defined as low. The co‐expression of CD68 and CD86 (CD68/CD86:+/+), ≥90.75/87.52, and co‐expression of CD68 and CD163 (CD68/CD163:+/+), ≥90.75/27.89, were defined as both high.

According to the cut points, 17 (26.6%), 10 (15.6%) and 38 (59.4%) patients were defined as having high CD68, CD86 and CD163 protein expression respectively. Furthermore, 8 (12.5%) and 10 (15.6%) patients were defined as having both high co‐expression of CD68 and CD86 and co‐expression of CD68 and CD163 respectively.

### 3.3 TAM biomarkers CD68, CD86 and CD163 correlation with Clinicopathological features in CRC patients

We studied whether the CD68, CD86 and CD163 expression level associated with clinicopathological features was potentially predictive of prognosis. The results found that CD68 protein expression was not significantly correlated with clinicopathological features (age, sex, tumour size, etc), patients with high CD86 expression showed a remarkably lower presence of poorer differentiation (*P* =.047) and more advanced tumour staging (TNM stage, *P* =.017), and not significantly associated with age, sex, tumour location, tumour size, T stage, lymph node metastasis and distant metastasis. Furthermore, patients with high CD163 expression showed a remarkably greater presence of larger tumour diameter and poorer differentiation (*P* =.04). The expression of CD68, CD86 and CD163 TAMs in CRC tissues was associated with different clinicopathological factors (Table 3).

**TABLE 3:** Expression of CD68, CD86 and CD163 protein in relation to clinicopathological parameters in colorectal cancer tissues

Variables	CD68		*P*‐value	CD86		*P*‐value	CD163		*P*‐value
	High	Low		High	Low		High	Low	
Age									
≤60	8	18	.529	6	20	.174	16	10	.771
>60	9	29		4	34		22	16	
Sex									
Male	9	26	.866	5	30	.746	21	14	.911
Female	8	21		5	24		17	12	
Tumour location									
Colon	9	19	.373	5	23	.664	14	14	.178
Rectal	8	28		5	31		24	12	
Tumour size (cm)									
<3	3	9	.892	4	8	.061	3	9	.007
≥3	14	38		6	46		35	17	
Differentiation									
Well/moderate	12	36	.624	10	38	.047	25	23	.040
Poor	5	11		0	16		13	3	
T stage									
T1‐T2	5	15	.849	4	16	.516	12	8	.945
T3‐t4	12	32		6	38		26	18	
TNM stage									
Ⅰ	5	13	.880	4	14	.017	11	7	.816
Ⅱ	3	11		1	13		9	5	
Ⅲ	7	20		2	25		16	11	
Ⅳ	2	3		3	2		5	2	
Lymph node metastasis									
No	9	24	.894	6	27	.561	16	10	.771
Yes	8	23		4	27		22	16	
Distant metastasis									
No	16	44	.942	8	52	.051	36	24	.693
Yes	1	3		2	2		2	2	

Note

*P*‐values were estimated by Chi‐squared test.

### 3.4 Correlation of CD86 protein expression and CD163 protein expression

The correlation of CD86 protein expression and CD163 protein expression is shown in Table 4. The level of CD86 protein expression showed a negative correlation with CD163 protein expression (r = −0.345,*P* =.005). The counts of CD163^+^ cells at the tumour area, but normally higher than that of CD86^+^ cells.

**TABLE 4:** Association of CD86 protein expression with CD163 protein expression

CD86	CD163			r	*P*‐value
	High	Low	Total no.		
High	2	9	11	−0.345	.005
Low	36	17	53		
Total no.	38	26	64		

Note

*P*‐values were measured by Fisher's exact test

### 3.5 Prognostic impact of TAMs biomarkers CD68, CD86 and CD163 expression in CRC

**FIGURE 3:**
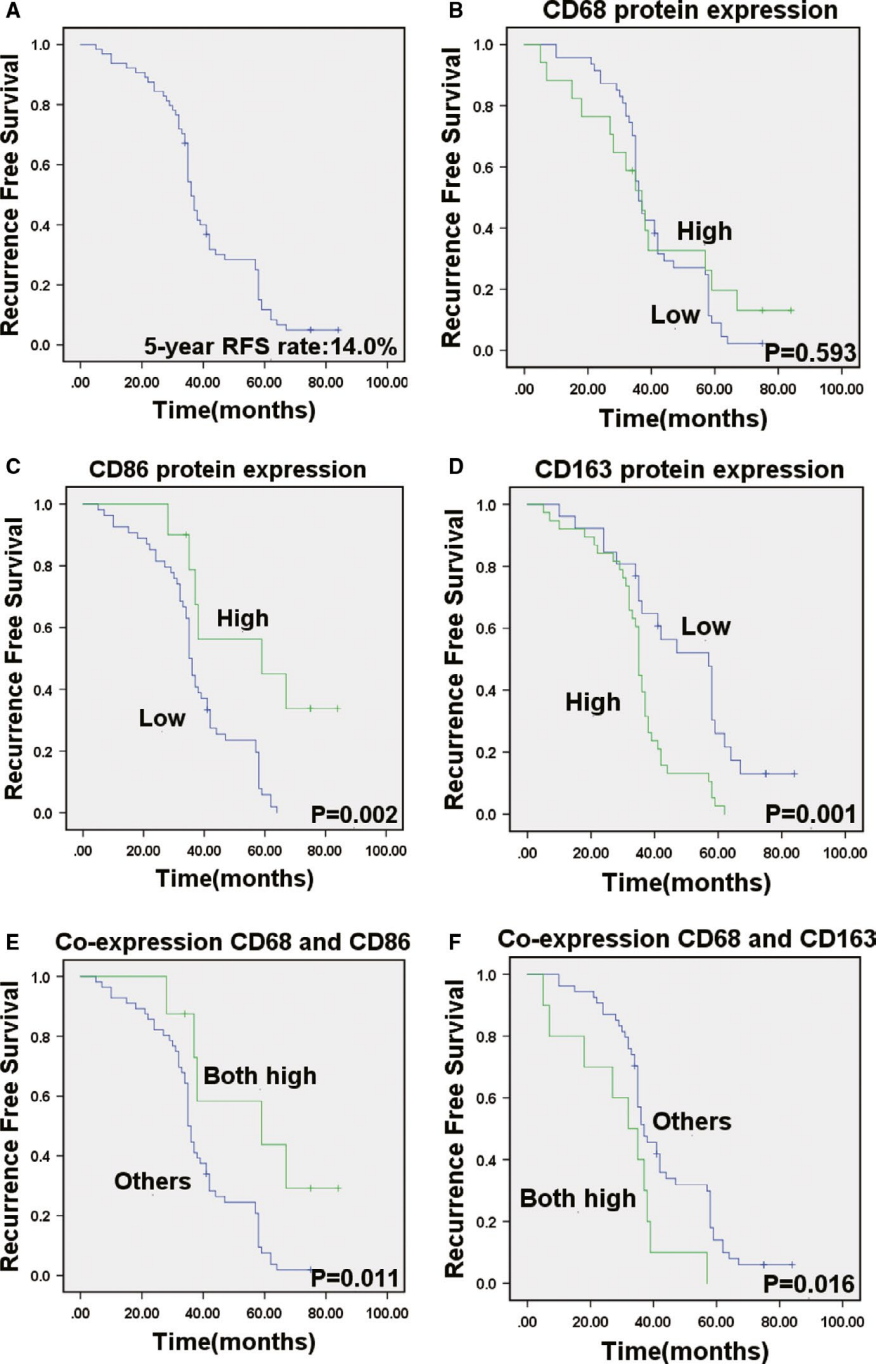
Aberrant CD68, CD86 and CD163 expression in tumours illustrates the prognosis in colorectal cancer patients for RFS. A. Kaplan‐Meier survival curves showed RFS in 64 patients. B. High CD68 expression is not associated with overall survival in CRC patients. C‐D. High expression of CD86 and low expression of CD163 are associated with favourable prognosis in human colorectal cancer samples. E. High co‐expression of CD68 and CD86 is associated with favourable prognosis. F. High co‐expression of CD68 and CD163 is associated with poor prognosis in CRC. The *P*‐value was obtained using the log‐rank test of the differences

**FIGURE 4:**
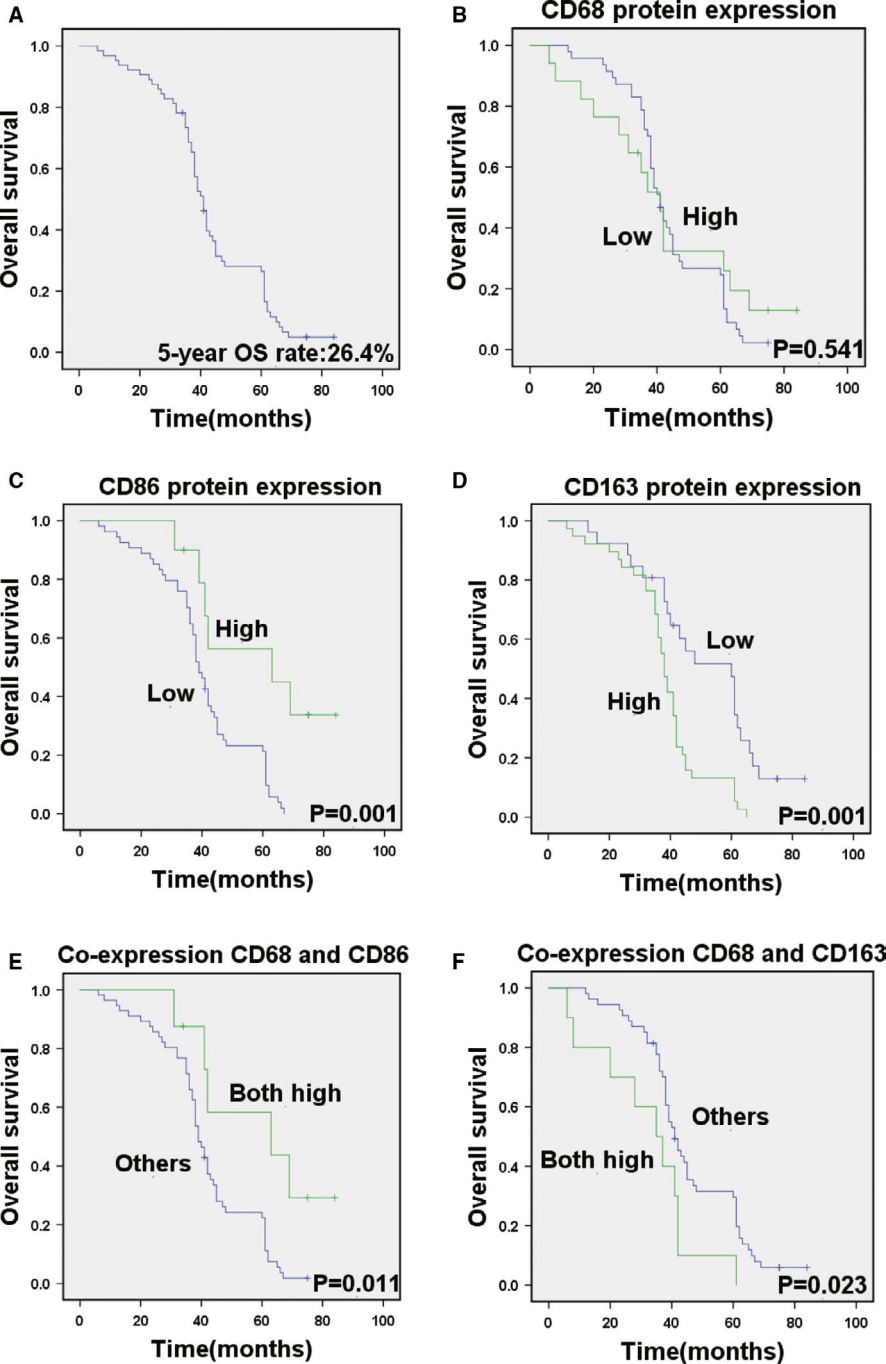
Aberrant CD68, CD86 and CD163 expression in tumours illustrates the prognosis in colorectal cancer patients for OS. A, Kaplan‐Meier survival curves showed OS in 64 patients. B, Cumulative OS differences between patients with high and low CD68 expression. High CD68 expression is not associated with overall survival in CRC patients. C‐D, Cumulative OS differences between patients with high and low CD86 and CD163 expression. E, Survival curves of patients with primary CRC are associated with high or low co‐expression of CD68 and CD86 and co‐expression of CD68 and CD163. The *P*‐value was obtained using the log‐rank test of the differences

To detect the prognostic impact of TAMs, we compared OS and RFS in patients with different expression of CD68, CD86 and CD163. CD68 protein expression was not a remarkable prognostic biomarker for RFS (*P* =.593) and OS (*P* =.541) (Figure 3B, Figure 4B). Kaplan‐Meier survival analysis found that there was a remarkable correlation between high CD86 expression and reduced RFS (*P* =.002) (Figure 3C). RFS was shorter in patients with low expression levels of CD86, whereas it was longer in those patients with high levels of CD86 expression. There was also a remarkable association between low CD86 expression and shorter OS (*P* =.001) (Figure 4C). CD86 expression status remarkably separates the OS of patients. In addition, RFS was shorter in patients with high expression levels of CD163, whereas it was longer in those patients with low levels of CD163 expression (*P* =.001) (Figure 3D). Simultaneously, there was a remarkable association between high CD163 expression and shorter OS (*P* =.001) (Figure 4D). Then, patients with both high co‐expression of CD68 and CD86 had significantly better RFS (*P* =.011) and OS (*P* =.011) than those with others (Figure 3E and Figure 4E). Patients with both high co‐expression of CD68 and CD163 had significantly worse RFS (*P* =.016) and OS (*P* =.023) than those with others (Figure 3F and Figure 4F).

### 3.6 CD86 protein expression and co‐expression of CD68 and CD163 TAMs as independent prognostic factor for RFS and OS

We assessed the Kaplan‐Meier survival of RFS and OS for tumour size, tumour differentiation, T stage, TNM stage, lymph node station and M stage (Figure S1 and S2). The results showed that the RFS was correlated with tumour size (*P* =.004), tumour differentiation (*P* =.011), T stage (*P* =.017), TNM stage (*P* =.023), lymph node metastasis (*P* =.019), CD86 protein expression (*P* =.002), CD163 protein expression (*P* =.001), co‐expression of CD68 and CD86 (*P* =.011) and co‐expression of CD68 and CD163 (*P* =.016) (Table 1). For these factors included in the Cox's multivariate analyses, CD86 protein expression (*P* =.007) and co‐expression of CD68 and CD163 (*P* =.001) were independent prognostic factor remarkably associated with RFS. In addition to CD86 protein expression and co‐expression of CD68 and CD163 status, statistically remarkable clinicopathological features that were associated with RFS were TNM stage (*P* =.001) and tumour differentiation (*P* =.010). Table 5 summarizes the results from the Cox proportional hazards analysis for RFS. Univariate analyses showed that the OS was correlated with tumour size (*P* =.003), tumour differentiation (*P* =.019), T stage (*P* =.008), TNM stage (*P* =.006), lymph node metastasis (*P* =.006), CD86 protein expression (*P* =.001), CD163 protein expression (*P* =.001), co‐expression of CD68 and CD86 (*P* =.011) and co‐expression of CD68 and CD163 (*P* =.023) (Table 2). In a multivariate Cox regression analysis, a low level of CD86 expression was predictive of decreased OS (*P* =.004), while a high co‐expression of CD68 and CD163 was predictive of reduced OS (*P* =.001). Moreover, TNM stage (*P* <.001) and tumour differentiation (*P* =.016) were also identified as independent prognostic factors. Table 6 summarizes the results from the Cox proportional hazards analysis for OS.

**TABLE 5:** Multivariate Analysis of significant prognosis factors for RFS in patients with colorectal cancer

Variables	Hazard ratio	95% CI	*P*‐value
TNM stage	0.111	0.032‐0.385	.001
Differentiation	0.435	0.232‐0.817	.010
CD86 protein expression	3.777	1.431‐9.968	.007
Co‐expression of CD68 and CD163	0.265	0.124‐0.567	.001

Note

*P*‐values were acquired by Cox proportional hazards analysis

**TABLE 6:** Multivariate Analysis of significant prognosis factors for OS in patients with colorectal cancer

Variables	Hazard ratio	95% CI	*P*‐value
TNM stage	0.091	0.026‐0.320	.000
Differentiation	0.461	0.246‐0.865	.016
CD86 protein expression	4.098	1.551‐10.832	.004
Co‐expression of CD68 and CD163	0.264	0.124‐0.566	.001

Note

*P‐*values were obtained by Cox proportional hazards analysis.

## 4 DISCUSSIONS

Colorectal cancer is the third primary cause of cancer death in the world. The TNM stage is generally known as the major prognostic factor, but it is not precise.^18^ Cancer biomarkers are substances or molecules objectively detectable in cells, body fluids or tissues that manifested the existence of cancer or the survival. Therefore, we hope to confirm a biomarker which is most correlated with CRC prognosis.

Despite the gigantic progress in the deconvolution of the immune infiltrate in tumour,^19^ the value of TAM‐derived signals for clinical prognosis is far from being comprehended. It is extensively confirmed that the immune system acted as a pivotal part in cancer development and progression,^14^ and macrophage closely related to outcomes of disease.^20^, ^21^ In addition, TAMs are one of the most dynamic immune cells in CRC, which are abundantly associated with the occurrence and development of cancers.^22^, ^23^ The ratio of M1/M2 macrophages has been considered to define these cells as either pro‐inflammatory or anti‐inflammatory.^24^ In specific stages, different subpopulations of TAMs have professional functions, yet they enhance growth with an inflammatory mutagenic environment at the prime period.^25^ During the progression phase, the primary function of M2 macrophages arouses angiogenesis, promotes tumour cell migration and invasion and suppresses anti‐tumour immunity. In most solid tumours, high‐density macrophages infiltration has been related to evidently poor prognosis.^11^, ^26^, ^27^ However, studies assessing the remarkableness of prognosis in different subtypes of TAMs infiltration in CRC remain controversial.^28^, ^29^, ^30^

We used IHC and IF to research the TAMs densities of tumour tissues and to confirm the correlation among TAMs, clinicopathological parameters and prognosis. Of the 64 cases, only 10 cases (15.6%) had a high level of CD86 expression. Although the numbers of patients were the limitation, it still illuminated the association between the expression level of CD86 and clinicopathological factors, and CRC prognosis. The research found that low CD86 expression was significantly correlated with clinicopathological characteristics including tumour differentiation or TNM stage, and high CD86 expression was a favourable predictor for RFS and OS. Patients with high CD163 expression were remarkably correlated with clinicopathological characteristics including tumour size or tumour differentiation and high CD163 expression was an adverse predictor for RFS and OS. Furthermore, CD86 protein expression level had a negative correlation with CD163 protein expression in CRC.

The traditional method of TAMs analysis was based individually on CD68 expression.^29^, ^31^, ^32^ In CRC, little researches implemented double‐IHC staining for analysing different subsets of TAMs, while the multitude used single‐IHC staining against M1 or M2 antigens.^33^, ^34^ We used 3 markers to identify TAMs; CD68, CD86 and CD163 were performed with pan‐macrophage markers, M1 macrophage markers and M2 macrophage markers respectively. We implemented double‐IHC staining for analysing different subsets of TAMs. Specially, we stained CD68 with green fluorescence and stained CD86 or CD163 with red fluorescence in the tumour tissues by IF co‐localization assay. These are little researches carried out. Previously, some studies found that M2 macrophages (CD163^+^) were related to poorer OS and DFS/RFS.^12^, ^31^ Moreover, low existence of CD86^+^ TAMs and high presence of CD206^+^ TAMs were outstandingly related with invasive tumour phenotypes and with poorer overall survival (OS) as well as reduced time to recurrence.^35^ And high CD163/CD68 ratio was closely associated with aggressive phenotype and poor prognosis in CRC.^34^ Viktor H. Koelzer et al suggested that high CD163^+^ TAMs infiltration predicted lower tumour grade, less lymph node metastasis and better prognosis.^36^ The significance of prognosis in M1 like or M2 like of TAMs infiltration in CRC remains controversial. In our study, univariate and multivariate analysis found that expression of CD86 is an independent prognostic factor for RFS and OS and high expression of CD86 TAMs was significantly associated with better RFS and OS. And we also identified expression of CD86 TAMs and expression of CD163 TAMs as a significant prognostic biomarker. However, multivariate analysis found that expression of CD163 TAMs could not significantly predict prognosis. As an improvement, we detected the co‐expression of CD68 and CD163 as the proportion of M2 TAMs in total TAMs. The results showed that co‐expression of CD68 and CD163 TAMs was a better account for the prognostic factor than expression of CD163 TAMs and traditional clinicopathological high‐risk factors. Furthermore, univariate and multivariate analysis found that TNM stage and tumour differentiation are independent prognostic factors for RFS and OS.

In conclusion, we researched prognostic remarkable of different subtypes of TAMs (pan‐macrophage marker: CD68; M1 macrophage marker: CD86 and M2 macrophage marker: CD163). The results found that strong CD86 expression in primary CRC tumour was correlated with tumour differentiation, TNM stage and better RFS and OS of CRC patients, and strong CD163 expression in primary CRC tumour was associated with tumour differentiation, tumour size and worse RFS and OS of CRC patients. The level of CD86 protein expression showed a negative correlation with CD163 protein expression. Moreover, CD86^+^ and CD68^+^CD163^+^ TAMs were defined as potential biomarkers of CRC development and progression. This disease has a high morbidity rate health problem in China, so a better analysis of the role of different subtypes of TAMs will be helpful for early diagnosis and treatment of CRC. Further experiments are conducted to study the mechanism of TAM in the CRC microenvironment and explored the drugs to promote polarization of TAM from M2 to M1 phenotype.

## CONFLICT OF INTEREST

The authors declare that there is no conflict of interest.

## AUTHOR CONTRIBUTIONS

Conception: Yu Kou, Baowei Han and Yunshuai Wang. Interpretation or analysis of data: Zhuoqun Li, Yan Li and Qidi Sun. Preparation of the manuscript: Shengnan Yang, Chen Hu and Yu Kou. Revision for important intellectual content: Zhuoqun Li, Yu Kou and Qidi Sun. Supervision: Huijie Gu, Huangjian Wang and Hairong Xu.

## Supporting information

Fig S1

Fig S2

Supplementary Material

## Data Availability

Date is available at the Scandinavian Journal of Immunology's website
