# Supplementary figures and images for: Prognostic value and predictive biomarkers of phenotypes of tumour‐associated macrophages in colorectal cancer
Source: Scand J Immunol. 2022 Jan 10;95(4):e13137. doi: 10.1111/sji.13137 (PMC9286461; doi:10.1111/sji.13137)

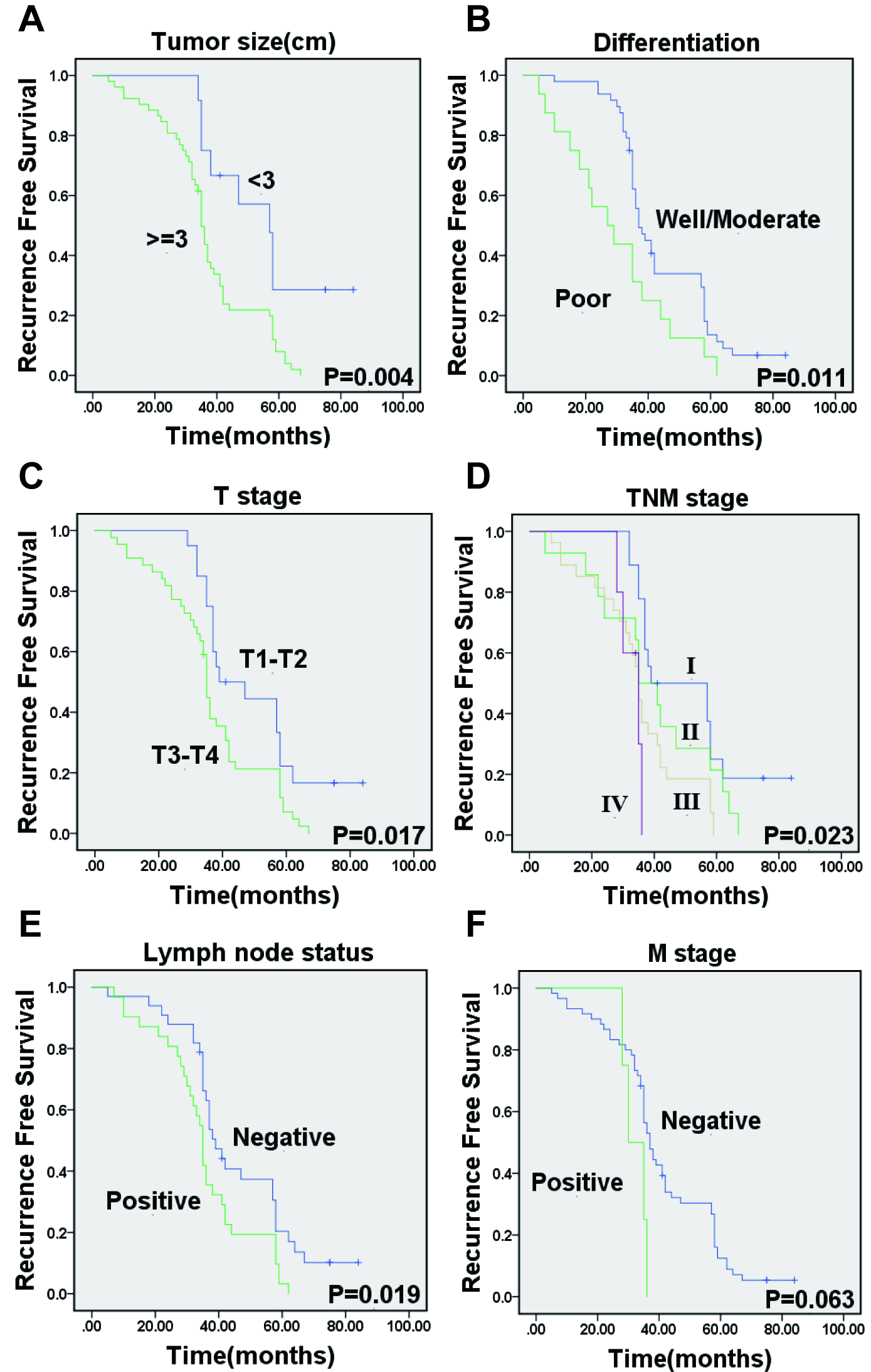

Supplement: Supplementary file 1 — Fig S1 [file SJI-95-0-s001.tif]

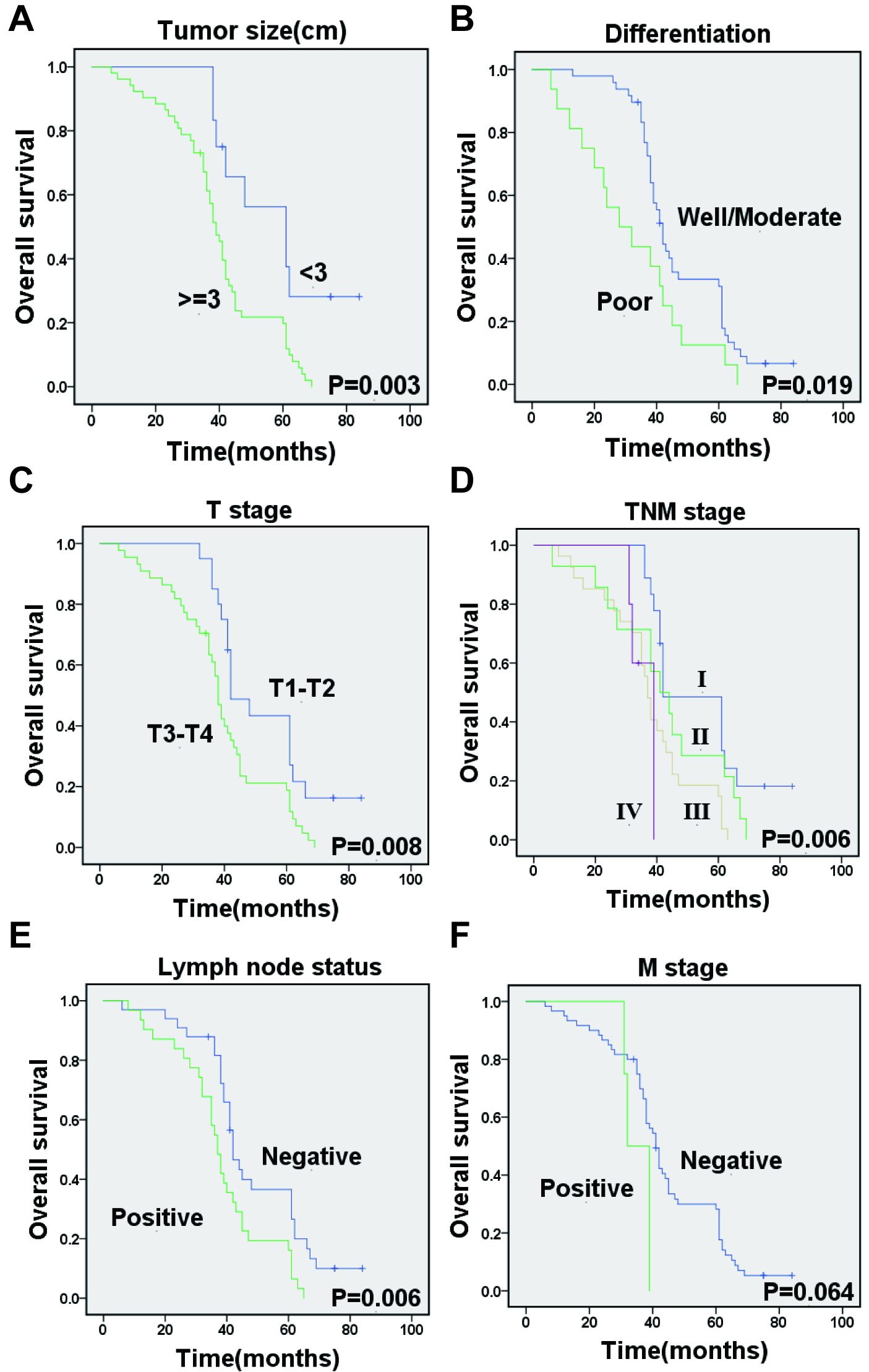

Supplement: Supplementary file 2 — Fig S2 [file SJI-95-0-s003.tif]
