# Supplementary material for: Prognostic value and predictive biomarkers of phenotypes of tumour‐associated macrophages in colorectal cancer
Source: Scand J Immunol. 2022 Jan 10;95(4):e13137. doi: 10.1111/sji.13137 (PMC9286461; doi:10.1111/sji.13137)
Supplement: Supplementary file 3 — Supplementary Material [file SJI-95-0-s002.docx]

Figure S1 Kaplan-Meier survival curves showed RFS of clinicopathological factors in CRC. A. Tumour size; B. Tumour differentiation; C. T stage; D. TNM stage; E. Lymph node status; F. M stage. The P-value was obtained using the log-rank test of the differences.

Figure S2 Kaplan-Meier survival curves showed OS of clinical and pathologic dates in CRC. A. Tumour size; B. Tumour differentiation; C. T stage; D. TNM stage; E. Lymph node status; F. M stage. The P-value was obtained using the log-rank test of the differences.
